# SARS–CoV-2 Immuno-Pathogenesis and Potential for Diverse Vaccines and Therapies: Opportunities and Challenges

**DOI:** 10.3390/idr13010013

**Published:** 2021-02-04

**Authors:** Andrew R. McGill, Roukiah Kahlil, Rinku Dutta, Ryan Green, Mark Howell, Subhra Mohapatra, Shyam S. Mohapatra

**Affiliations:** 1 Department of Veterans Affairs, James A. Haley Veterans Hospital, Tampa, FL 33612, USA; armcgill@usf.edu (A.R.M.); roukiah@usf.edu (R.K.); rinku@usf.edu (R.D.); rjgreen@usf.edu (R.G.); mhowell1@usf.edu (M.H.); 2 Department of Internal Medicine, Morsani College of Medicine, University of South Florida, Tampa, FL 33612, USA; 3 Department of Molecular Medicine, Morsani College of Medicine, University of South Florida, Tampa, FL 33612, USA; 4 Pharmacy Graduate Programs, Taneja College, MDC30, 12908 USF Health Drive, Tampa, FL 33612, USA

**Keywords:** SARS-COV-2, COVID-19, immunopathogenesis, therapeutics, vaccines

## Abstract

Severe Acute Respiratory Syndrome Coronavirus-2 (SARS-CoV-2) is a novel coronavirus that emerged from Wuhan, China in late 2019 causing coronavirus disease-19 (COVID-19). SARS-CoV-2 infection begins by attaching to angiotensin-converting enzyme 2 receptor (ACE2) via the spike glycoprotein, followed by cleavage by TMPRSS2, revealing the viral fusion domain. Other presumptive receptors for SARS-CoV-2 attachment include CD147, neuropilin-1 (NRP1), and Myeloid C-lectin like receptor (CLR), each of which might play a role in the systemic viral spread. The pathology of SARS-CoV-2 infection ranges from asymptomatic to severe acute respiratory distress syndrome, often displaying a cytokine storm syndrome, which can be life-threatening. Despite progress made, the detailed mechanisms underlying SARS-CoV-2 interaction with the host immune system remain unclear and are an area of very active research. The process’s key players include viral non-structural proteins and open reading frame products, which have been implicated in immune antagonism. The dysregulation of the innate immune system results in reduced adaptive immune responses characterized by rapidly diminishing antibody titers. Several treatment options for COVID-19 are emerging, with immunotherapies, peptide therapies, and nucleic acid vaccines showing promise. This review discusses the advances in the immunopathology of SARS-CoV-2, vaccines and therapies under investigation to counter the effects of this virus, as well as viral variants.

## 1. Introduction

The recent emergence of Severe Acute Respiratory Syndrome Virus-2 (SARS-CoV-2) in December of 2019 in Wuhan, China causing coronavirus infectious disease-2019, referred to as COVID-19, reaffirms the clinical significance of zoonotic coronaviruses. Before the 2003 SARS-CoV-1 epidemic, the virology of Coronaviridae was poorly studied. Along with SARS-CoV-1 and Middle Eastern Respiratory Syndrome Coronavirus (MERS-CoV), recently, SARS-CoV-2 has become the third coronavirus to reach epidemic, and subsequently pandemic status. The emergence of this novel coronavirus has a significant implication to the global population, given that cross-reactive immunity from other viral exposure is unlikely, indicating that the vast majority of people could be susceptible to infection [1]. SARS-CoV-2 has been characterized as causing severe respiratory distress that can lead to pneumonia and acute respiratory distress syndrome (ARDS), as well as clotting abnormalities and stroke [2,3]. These severe viral infection manifestations seem to significantly burden the elderly and those with underlying conditions, although severe cases have been seen in the young and healthy as well [1]. Prominent risk factors for COVID-19 include obesity, which is highly prevalent in the USA (42% in 2017–2018), heart disease, pulmonary disease, and diabetes [4,5,6,7]. These risk factors implicate a majority of the United States population with increased susceptibility to severe disease from COVID-19. One unique feature of the SARS-CoV-2 pandemic is its high asymptomatic carrier rate (up to 46%), which has been an underlying factor for this coronavirus’s unprecedented spread across the globe [8,9]. A meta-analysis suggests that SARS-CoV-2 infection has an incubation period of 5–7 days (with an average of 5.7 days), which can extend up to 14 days for those with previously mentioned comorbidities who are particularly at risk for cytokine storm syndrome (CSS), organ damage, and thrombosis in response to SARS-CoV-2 infection. The complexity of this viral infection warrants early interventions using combinatorial methods to successfully combat COVID-19, since no single therapy has been shown to be fully effective [10]. Hence, a plethora of vaccines, prophylactics, and treatment modalities are being intensely investigated against COVID-19 and will be reviewed herein.

Coronaviruses belong to the family Coronaviridae in the order Nidovirales. They are large, enveloped, positive-sense-single-stranded RNA (+ssRNA) viruses, having the most extensive viral RNA genome, ranging from 27 to 32 kb. The capped and polyadenylated +ssRNA genome is akin to mRNA, infectious upon entry into the cell, ready for translation at multiple open reading frames (ORFs), to begin the viral lifecycle. The SARS-CoV-2 RNA genome contains 10 ORFs. ORF1ab encodes for the viral replicase polyprotein, which is further processed via protease into 16 distinct non-structural proteins (NsPs) [11]. ORF2-10 encodes for the viral structural and auxiliary proteins responsible for forming the viral coat and packaging of the RNA genome [11]. We have recently begun to understand the roles of some of the underdefined NsPs outside of viral replication, specifically, how NsP1, NsP3, NsP5, and ORF7a antagonize the host’s immune response, causing dysregulation resulting in immune escape of the virus. Despite the progress made, several questions relating to SARS-CoV2 remain to be answered, including how the virus gains access to host cells, how the virus antagonizes the host immune system, implications of the emerging variant strains of SARS-CoV-2, as well as therapeutic options and vaccines that are under development will be the focus of this review. 

## 2. Biology of SARS-CoV-2 Infection

### 2.1. Virus–Host Interaction and Viral Entry

The host range of a coronavirus can be attributed mainly to the binding affinity of the viral spike protein (S) with its cognate host cellular receptor(s). Like SARS-CoV-1, SARS-CoV-2 uses the type 1 membrane protein angiotensin-converting enzyme 2 receptor (ACE2) to access host cells. Notably, the S–ACE2 interaction is the target of many vaccination and therapeutic efforts, as the neutralization of S, particularly at the receptor-binding domain (RBD), would significantly hamper viral infectivity. In humans, ACE2 is highly expressed in nasal and colonic tissues [12,13,14]. ACE2 is further expressed throughout the body in the lungs, kidneys, heart, and intestines [15]. Interestingly, outside of respiratory infection, SARS-CoV-2 has also been detected in the fecal material and brainstems of infected patients and animals [16,17]. The primary role of ACE2 is the cleavage of host angiotensin II, which regulates normal blood pressure, specifically vasoconstriction [15]. Attachment and the cell membrane fusion of SARS-CoV-2 have been shown to downregulate the ACE2 receptor [18]. Importantly, with loss of catalysis of this receptor at the membrane, there is a reported increase in pulmonary inflammation and coagulation, which can be attributed to a higher free angiotensin II and ACE2 downregulation-associated effects [19]. Given that reduced ACE2 expression caused by SARS-CoV-2 is associated with inflammation and coagulation, patients with lower baseline levels of ACE2 may have a poorer prognosis from COVID-19, further diminishing the protective role of ACE2 in maintaining a balance between vasoconstriction and vasodilation [20]. However, other receptors such as CD147 and neuropilin-1 (NRP1) are under active investigation as potential receptors for SARS-CoV-2 as well [12,13,14,21,22,23]. The neurological activity of SARS-CoV-2 has also been observed, despite the lack of abundant ACE2 expression in the brain, which supports the possibility that SARS-CoV-2 may be capable of entering cells through more than one receptor [13,24,25]. 

The SARS-CoV-2 S protein binds to the host receptor via the S1 domain and then fuses to the host cell via the S2 domain. Host transmembrane protease serine 2 (TMPRSS2) cleaves of the S1/S2 region revealing the S2 domain, which subsequently fuses with the host cell membrane [24]. Additionally, TMPRSS2 & 4 have been identified as contributing to small intestine enterocytes’ productive infection, explaining SARS-CoV-2 RNA being detected in fecal matter [25]. Others have hypothesized the role of other proteases, such as furin, that could also serve as the necessary factor in priming the S protein for fusion. Analysis of the S protein of SARS-CoV-2 identified an insertion of a furin cleavage site (FCS) consisting of multiple basic amino acids at the S1/S2 site [26]. 

Interestingly, this correlates with FCS insertions in the hemagglutinin (HA) cleavage site of highly virulent influenzas that was cleaved by host furin protease [27,28]. This could, in part, help explain the infection of cell types that may lack TMPRSS2, as priming of the S protein for fusion is an essential part of the infection process. The results of the studies that are defining the attachment and fusion of SARS-CoV-2 to host cells will better inform the development of therapeutics that may antagonize these processes that are essential the viral life cycle. 

### 2.2. Potential Role of CD147

Along with ACE2, SARS-CoV-2 has also been shown to interact with CD147 (aka Basigin or EMMPRIN; Extracellular Matrix Metalloproteinase inducer), which is a transmembrane glycoprotein present on the host cell that may be labile for infection [22,29]. CD147 is found on various cell types such as epithelial cells, fibroblasts, brain tissue, and cancer cells. It has been proved to be a prognostic marker of tumor progression by inducing matrix metalloproteinases (MMP). Furthermore, this receptor is upregulated in models of traumatic brain injury (TBI) and ischemia [24,30,31]. Interestingly, in 2003 SARS-CoV-1, the N protein coprecipitated with CD147, raised the possibility of alternative pathways of interaction of viral structural components with this receptor [23]. In 2005, Chen et al. studied the functional role of HAb18G/CD147 in the invasion of the host cell by SARS-CoV-1 where the authors found the N protein of SARS-CoV-1 was bound to Cyclophilin A (CyPA). CyPA is needed for replication of coronaviruses, and thus, the inhibitor of CyPA, cyclosporine A, has been shown to exhibit broad spectral inhibition of Coronaviruses [28]. Additionally, with the help of HAb18G/CD147—antagonistic peptide (AP)—9 in HAb18G/CD147-expressing HEK293 cells, they showed the inhibition of SARS-CoV-1 binding [23,28]. Apart from SARS-CoV-1, CD147 also facilitates HIV-1 infection via CyPA association as well [32]. Considering alternative binding of S, although in silico approaches have shown the interaction of SARS-CoV-2 S with sialic acid bearing gangliosides and CD147, more data are required to understand and elucidate alternative receptor binding [22,33]. Recently, however, Wang et al. have demonstrated the co-localization of CD147 and S of the SARS-CoV-2. Using a anti-CD147 antibody, Meplazumab, led to the blocking of the CD147 receptor and inhibition of SARS-CoV-2 replication in a dose-dependent manner [29]. In an open-labeled, concurrent controlled add-on clinical trial, patients treated with Meplazumab (10 mg) recovered their normal lymphocyte count and elevated C-reactive protein (CRP) concentrations. Thus, the drug’s efficacy to treat SARS-CoV-2 patients with pneumonia carries potential without adverse effects [30]. Therefore, the application of humanized anti-CD147 antibody holds the potential for anti-viral treatment. Although the cellular, structural basis of the protein, and its role in TBI and cancer progression is known [31], this receptor’s functional role in SARS-CoV-2 is just starting to be understood, which has the utmost importance in the development of effective preventatives and therapeutics against COVID-19.

### 2.3. Role of NRP1

NRP1 is a type I transmembrane protein containing multiple domains that function as a coreceptor for numerous ligands, such as vascular endothelial growth factor, transforming growth factor-beta, and semaphorins [34]. NRP1 was first studied in the nervous system, where it was shown to have versatile roles in axon guidance, neuronal development, cell survival, angiogenesis, and migration [35,36]. Many of these observations investigating the roles of NRP1 came from cancer studies that use these pathways to upregulate tumoral angiogenesis. More recently, NRP1 has also been shown to have a relationship with the host immune system, acting as a marker of CD4+FoxP3+ regulatory T cells as well as a subset of tumor-infiltrating CD8 cells [37]. Furthermore, NRP1 was also recently shown to regulate secondary CD8 T Cell differential responses to viral infection, contributing to a memory phenotype [38]. Most recently, NRP1 has been described as a host receptor for SARS-CoV-2 attachment and subsequent entry, which has significant implications. Neurologic complications during COVID-19 have previously been described as originating from a surge of inflammatory cytokines, such as IL6, which damages the central nervous system [39]. While the systemic inflammatory response to SARS-CoV-2 may contribute factor to neuronal pathology, recent evidence suggests that NRP1 may contribute to direct damage to nervous system tissues by acting as a receptor for SARS-CoV-2. Recent work by Cantuti-Castelvetri et al. and Daly et al. show the importance of this receptor during the infection process, which has significant neurological considerations [40,41]. Furin protease cleaves the full-length S, exposing the S2 domain from S1. This cleavage produces a polybasic Arg-Arg-Ala-Arg carboxyl-terminal sequence on the S1 domain, which then conforms to a C-end rule motif that can bind to the NRP1 receptors [40]. Cells shown to express NRP1 were shown to be permissive to SARS-CoV-2 infection, with antibody blockade of NRP1 reversing this effect [40,41]. These data provide further insight into SARS-CoV-2 cell infectivity, which is still clearly being defined. The results presented in these studies are compelling, but further investigations from in vivo models or clinical samples are necessary to provide greater validity in determining this receptor’s role in SARS-CoV-2 infection. Importantly, these works provide another potential target for SARS-CoV-2 antiviral intervention and warrant more significant investigation.

## 3. Immune Response to SARS-CoV-2

**Figure 1:**
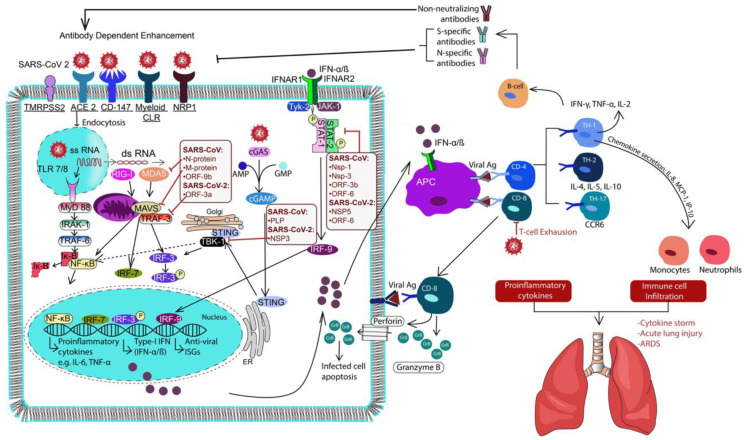
Schematic illustration overviewing Severe Acute Respiratory Syndrome Coronavirus-2 (SARS-CoV-2) infection mechanism and the host immune response post-infection. TMPRSS2, angiotensin-converting enzyme 2 receptor (ACE2) and CD147 play pivotal roles in the viral attachment and entry followed by post-infection and viral replication. Host immune defense begins sooner with the initiation of PRR signaling (TLRs, RLRs, CLRs and cGAS-STING) leading to activation of type I IFN along with proinflammatory cytokine. TLRs, Toll-like receptors; RLRs, RIG-1 like receptors; CLRS, C-lectin like receptors; cGAS-STING, cyclic GMP-AMP synthase-Stimulator of Interferon Genes; IFN, interferon.

The host immune system is the first line of defense against viral infections [42]. Viral pathogen-associated molecular patterns (PAMPs) and damage-associated molecular patterns (DAMPs) e.g., ATP, DNA, RNA, and ASC (aka PYCARD, often referred to as ASC: Apoptosis-associated speck-like protein containing a CARD), oligomers, are recognized by the innate immune cells through pattern recognition receptors (PRRs) [43,44]. This results in the activation of the antiviral interferon response, complement system, and other innate mediators meant to limit viral replication and spread. Antigen-presenting cells (e.g., dendritic cells and macrophages) uptake and present viral antigens to elicit an adaptive humoral and cellular response [45,46]. Typically, neutralizing antibodies and cytotoxic T-cells limit viral spread and target infected cells, respectively. Although the immune response usually clears the infection, it can be hampered by the viral gene products, leading to inadequate immune responses, as implicated by the viral NsPs, which will be covered in greater detail throughout this section [47,48]. A deregulated and overactive immune response resulting in excessive inflammation is a significant contributor to coronavirus-mediated lung damage and systemic pathology. Viral binding has implications in this pathology on its own, which can be associated with the downregulation of ACE2 as previously covered, as well as direct immune dysregulation caused by the virus itself [49]. Reviewed herein are the currently reported SARS-CoV-2 immune dysregulation and evasion mechanisms and protective anti-viral innate and adaptive immune responses, which can also be seen illustrated in Figure 1.

### 3.1. Immune Subversion by NsPs of SARS-CoV-2

The immune response to SARS-CoV-2 is currently an intense research area, as immuno-pathology has been shown to be a primary culprit of COVID-19 lethality. The viral NsPs play a significant role in developing the observed immuno-pathology of SARS-CoV-2 by dysregulating the anti-viral immune response. The first two open reading frames, ORF1a and ORF1b, of SARS-CoV-2 comprise about two-thirds of the viral RNA genome and encode for 16 NsPs, which antagonize the host immune response and replicate the viral genome. Between ORF1a and ORF1b lies a −1 frameshift, that results in the translation of two polypeptides (pp1a and pp1ab). Viral proteases NsP3 and NsP5 auto-cleave from the polypeptides using their Papain-Like-Protease (PLpro) and 3-chymotrypsin-like protease (3CLpro) domains, respectively. Interestingly, these domains observed in NsPs are conserved across Nidovirales. NsP3 proteolytically processes Nsp1 and 2, containing the XLXGG domain pattern for PLpro, whereas NsP5 proteolytically processes NsPs 4–16. Given that most proteolysis events occur via NsP5, this specific protease is often referred to as the Major Protease, or Mpro throughout the literature. These proteolytically processed NsPs have dedicated functionality to viral replication and host interferon antagonism. The antagonism of the type-1 IFN response can be attributed, in part, to the robust immuno-pathology observed in COVID-19. Recently, Gao et al. resolved the SARS-CoV-2 RNA Dependent RNA Polymerase (RdRp) crystal structure, showing NsP12 functioning as the major polymerase subunit, with NsP7 and 8 acting as cofactors [50]. It is important to note that these NsPs are proteolytically processed by NsP5, promoting this particular NsP as an attractive antiviral drug target.

Apart from their role as proteases, NsP3 and NsP5 along with NsP1 can be considered significant virulence factors of SARS-CoV-2 [11]. It has been observed in SARS-CoV-1 that these proteins are specifically related to the antagonism of innate immunity and type-1 IFN production, providing a means for immune escape. Previously, it has been demonstrated that NsP1 antagonizes host type-1 IFN production by degrading exogenous host mRNA and by interacting with the host 40 s ribosome, decreasing mRNA synthesis [51,52]. NsP3 is the largest of the NsPs and contains 16 domains. This protein interacts with viral structural, non-structural proteins, and host proteins as a scaffolding element to subvert host immune responses [53,54]. As previously discussed, a major domain of NsP3 is the PLpro, which is responsible for this protein’s protease activity. The PLpro domain can interact with IFN-β stimulating components, such as the formation of the TRAF3–TBK1 complex responsible for regulatory factor IRF3 production, which subsequentially activates IFN-β [55]. Moreover, NsP3 in NL63 coronavirus stabilizes MDM2, an E3 ubiquitin ligase, by the process of de-ubiquitination, which increases the rate of degradation of p53, suppressing T1-IFN signaling [56]. From these observations, further study into SARS-CoV-2 NsP3 as an attractive antiviral drug target is warranted, given its multiple roles during the viral life cycle. Similarly, NsP5 serves as an attractive drug target, given its 3CLpro domain activities in proteolytically processing the viral NsPs, including the RNA-dependent RNA polymerase. However, similar to NsP3, NsP5 has other interactions with the host that aim to subvert immune activation. It has been shown that NsP5 targets and cleaves NF-kappa-B essential modulator (NEMO) and STAT2, which suppresses Type 1-IFN production and inhibits the transcription of interferon-stimulated genes (ISGs), respectively [57,58]. NsP5 cleaves STAT2 by targeting two regions on the P1 position of the protein with glutamine residues, an observation used to design biomimetic drugs against this NsP [57,59]. SARS-CoV-2 NsP5 was found to be a relatively more potent inhibitor of IFN-β than SARS-CoV-1 NsP5 by targeting IFN-stimulated response elements (ISRE), NF-kb signaling, K63-linked ubiquitination of RIG-I, and its interaction with its E3 ligase TRIM25 [60]. Moreover, SARS-CoV-2 NsP5 impaired IFN-mediated STAT-1 phosphorylation by increasing its colocalization with LC3B and hence increasing its autophagic degradation. As a result, NsP5 overproduction decreased viral replication, IFN production, and ISGs. SARS-CoV-2 NsPs were recently found to inhibit type-I IFN signaling via different mechanisms [61]. For example, NsP6 and NsP13 inhibit IRF-3 phosphorylation by binding TBK-1, while ORF-6 inhibits IFN production and signaling via IRF-3 nuclear translocation. Furthermore, SARS-CoV-2 NsP1, NsP6, NsP13, ORF3a, M, ORF7a, and ORF7b suppress IFN-I signaling by inhibiting STAT1/STAT2 phosphorylation. Interestingly, SARS-CoV-2 NsP1 and NsP6 were found to be more efficient in inhibiting IFN-I than SARS-CoV-1 or MERS. In a different study, SARS-CoV-2 NSP1, NSP3, NSP12, NSP13, NSP14, ORF3, ORF6, and M protein inhibited IFN-β promoter activation induced by Sendai virus [62]. The c-terminus of ORF-6 is necessary to inhibit IFN-I by inhibiting the nuclear translocation of phospho-STAT1 [62]. Understanding the roles of the NsPs during SARS-CoV-2 infection and their impact on the overall immune response is key to subverting the potentially devastating health outcomes from this virus. The mounting evidence elucidating the roles of the SARS-CoV-2 NsPs illustrate them as potentially potent anti-viral drug targets; however, more significant characterization and inhibition studies of these specific NsPs are needed.

### 3.2. Innate Immune Activation

Following viral entry and replication by SARS-CoV-2, lung cells undergo pyroptosis, leading to inflammation, the release of IL-1β, and the recruitment of immune cells to the site of infection [63]. PRRs bind PAMPs (e.g., viral RNA) and DAMPs to initiate PRR signaling cascade that activates the transcription of type I IFN-α/β and proinflammatory cytokines. TLRs, RLRs, C-lectin like receptors (CLRs), and cyclic GMP-AMP synthase-Stimulator of Interferon Genes (cGAS-STING) are four classes of PRRs that are involved in the immune response to Coronaviruses [64]. The importance of PRRs’ signaling and viral immune evasion mechansims in SARS-CoV-2 infections remain to be uncovered. However, previous studies show that PRRs’ signaling is important to clear SARS-CoV-1 and MERS infections [57,64]. In this subsection, we will discuss PRRs’ signaling and how Human Coronaviruses (hCoVs) can target PRR signaling to delay the anti-viral IFN response [65].

To date, thirteen TLRs have been identified in mice and humans with TLRs 1–6 found on the plasma membrane while TLR 3, 7–9 are located within endosomes [66]. In vivo studies show that MyD88, TRIF, TLR3, and TLR4-deficient mice show a worsened lung pathology and increased susceptibility to SARS-CoV-1 infection [67,68]. The TLR signaling adapter protein Ticam2 is required for a protective anti-viral immune response as Ticam2-deficient mice show more severe pulmonary pathology upon SARS-CoV-1 infection than wild-type mice [69]. Furthermore, viral SARS-CoV-1 NsP3 with the papain-like protease domain was shown to directly inhibit the TLR7 signaling pathway by removing Lys63-Linked Polyubiquitination of TRAF3 and TRAF6, resulting in reduced viral detection by the immune system and interferon induction [70]. Interestingly, the Coronavirus Porcine Epidemic Diarrhea Virus (PEDV) induced NF-kB signaling through TLR2, TLR3, and TLR9 in response to infection. Specifically, it was found that the PEDV nucleocapsid protein (N) could activate NF-κB with the central region of N being essential for NF-κB activation [71]. The inhibition of NF-kB was shown to increase survival in SARS-CoV-1 infected mice, showing how this process of immune dysregulation results in aberrant inflammation, with possible contribution to TLR inhibition and induction by viral proteins [72].

The SARS-CoV-2 N protein antagonizes the type I interferon response by suppressing phosphorylation and nuclear translocation of STAT1 and STAT2 [73]. Moreover, SARS-CoV-2 membrane (M) protein antagonizes the type I and III IFN response by physically interacting with RIG-I/MDA-5, thus preventing signaling [74]. ORF-9b protein causes the degradation of MAVS, TRAF3, and TRAF6, reducing the host IFN response [75]. Similar to SARS-CoV-1, SARS-CoV-2 ORF9b localizes to the outer mitochondrial membrane and binds TOM-70 (an adapter involved in the RIG-I/MAVS signaling complex). This impairs the T1-IFN response and can be reversed by TOM-70 overexpression [76].

The cyclic GMP-AMP synthase (cGAS)-STING system is a cytoplasmic DNA sensor commonly targeted by hCoVs. In the presence of an infection, the cell membrane-bound, monomeric cGAS binds the foreign DNA to form liquid droplets and synthesizes cGAMP from AMP and GMP [77,78]. Then, cGAMP binds the stimulator of interferon genes (STING) protein, leading to its translocation from the ER to the Golgi apparatus and then to the perinuclear microsomes. During translocation, a complex is formed between STING, TBK1 kinase, and IRF3, where TBK1 phosphorylates IRF3, leading to its nuclear translocation and the activation of type-I IFN response. Even though STING is best known for its role in combating bacterial and DNA viral infection, evidence shows that it plays a role in tackling RNA viruses as well. RNA virus infection increases the activation of STING, and its deficiency increases the replication of positive and negative-strand RNA viruses in vivo and in vitro [79,80,81,82]. Sun et al. showed the significance of cGAS-STING signaling in SARS-CoV-1 infection as hCoV NL63 and SARS-CoV-1 PLP reduced STING dimerization in infected cells [64]. The expression of membrane-anchored PLP domain from either virus inhibits STING-mediated IRF3 activation. Although SARS-CoV-2 PLpro has 83% sequence identity to SARS-CoV-1 PLP, it preferentially binds the ubiquitin-like IFN-stimulated gene 15 protein (ISG15) with high affinity, leading to the cleavage of ISG15 from IRF3, abrogating IFN response [83]. Consequently, a SARS-CoV-2 PLPro inhibitor (e.g., GRL-0617) was proposed as a therapeutic strategy by inhibiting viral replication, cytopathic effect, and sustaining IFN signaling.

Myeloid C-type lectin receptors (CLRs) are glycan-binding receptors that recognize glycans-coating pathogens to elicit various immune responses including phagocytosis, antigen presentation, and cytokine secretion [84]. Myeloid CLRs contain intracellular signaling domains: immunoreceptor tyrosine-based activation motif (ITAM), hemi-ITAM and immunoreceptor tyrosine based inhibitory motif (ITIM) [85]. Both ITAM and hemi-ITAM containing CLRs activate NF-κB, NFAT, and AP-1 transcription factors, leading to immune activation. Glycan moieties in viral glycoproteins play an essential role in host–viral interactions, viral antigenicity, and internalization. The (S) glycoprotein of SARS-CoV-1 and SARS-CoV-2 binds to the ACE2 receptor expressed on the host cells, leading to viral entry. Additionally, two CLRs (DC-SIGN and L-SIGN) are involved in SARS-CoV-1 pathology by offering alternative viral entry routes [86,87]. The serum CLR mannose-binding lectin (MBL) binds SARS-CoV-1 (S) glycoprotein, therefore inhibiting its binding to the CLR DC-SIGN without influencing its binding to ACE-2 [88]. A recent transcriptomic study shows that smoking increases ACE2 and DC-SIGN expression in alveolar type-2 (AT2) and monocytes/DCs, respectively [89]. Therefore, smoking could predispose SARS-CoV-2 infection by enhancing viral entry and promoting an abnormal inflammatory response. CLRs play a critical role in pathologic inflammation and cytokine storm in MERS infection [90]. MERS replication significantly increased the expression of CLR macrophage-inducible calcium-dependent lectin receptor (Mincle). The antibody blockage or siRNA-mediated knockdown of Mincle significantly decreased the cytokine levels of IL-6, RANTES, IP-10, and IFN-γ, abrogating the pathologic proinflammatory response. Understanding the initial reaction to the virus at the innate level could better help inform therapeutic development that may negate viral infection by bolstering innate immune responsiveness. Greater investigation into these innate mechanisms discussed in this section is warranted and could serve to outline differences in the acute response to the virus, which varies significantly from patient to patient.

### 3.3. Adaptive Immune Responses

APCs (DCs and macrophages) connect the immune system’s innate and adaptive arm by presenting foreign antigens to prime T and B-cell response [91,92]. To evade immune elimination, coronaviruses hinder DC maturation, antigen presentation, or skew the resulting T-cell response from a protective TH1 response to a TH2 response [46]. In vitro SARS-CoV-1 and CoV-2 infection of DCs and macrophages causes an increase of proinflammatory cytokines (IL-6, IL-8, MCP-1, MIP-1, TNF-α) and a decrease in IFN response and IL-12p40 [93,94,95,96]. The inspection of lymphoid organs of COVID-19 post-mortem cases shows that SARS-CoV-2 infects tissue-resident CD169+, ACE2+ macrophages, and subsequently stimulate IL-6 secretion [97]. The upregulated cytokine response in the absence of an effective anti-viral immunity contributes to the development of inflammatory pathology, including ALI and CSS. Importantly, IL-6, along with TNF-α, was shown to be a marker of severe COVID-19 disease and increased risk of death [98]. This proposed cytokine storm can be attributed to activated macrophages, particularly alveolar macrophages, which may contribute to lung damage [99].

The bronchoalveolar lavage fluid isolated from severe COVID-19 cases shows that the alveolar space contains alveolar macrophages and T-cells without neutrophils [100]. Despite the lack of ACE-2, alveolar macrophages had SARS-CoV-2 transcripts indicating that it might be able to infect these cells, leading to chemokine secretion and T-cell recruitment. Consequently, recruited T-cells secrete IFN-γ which activates alveolar macrophages, forming a positive feedback loop of inflammation. SARS-CoV-2 neutralizing antibodies (NAbs) were detected in the plasma 10–15 days after the onset of symptoms and did not cross-react with SARS-CoV-1 [101]. However, it has been reported that in about 10% of severe cases of SARS-CoV-2, the infection had neutralizing IgG autoantibodies against interferons, specifically for IFN-α, IFN-ω, and the other type 1 IFNs [102]. These autoantibodies negate the ability of IFNs to block SARS-CoV-2 replication and were not found in mild SARS-CoV-2 or asymptomatic cases. In addition, certain severe cases of SARS-CoV-1 and CoV-2 were successfully treated using convalescent sera, confirming the protective nature of these Abs by neutralizing the S-glycoprotein and inhibiting viral entry [103,104].

Furthermore, SARS-CoV-2 convalescent sera did not show an antibody response to mPro NsP5, indicating that it may not be an antigen for humoral immunity [105]. Antibodies specific to SARS-CoV-2 (RBD) and (N) proteins were detected in follow-up patients where the sera’s neutralizing ability was correlated to the levels of anti-RBD IgG but not to anti-N IgG. Elderly patients showed significantly higher levels of NAbs and spike-binding antibodies. NAb titers were found to correlate with C-reactive protein levels positively and inversely correlate with lymphocyte counts [79]. Despite the protective nature of anti-S antibodies, Hoepel et al. have recently found that stimulating primary alveolar macrophages with patient-derived anti-S IgG immune complexes combined with poly I:C leads to a robust inflammatory response characterized by high levels of IL-1β, IL-6, and TNF-a [106].

CD4 and CD8 T-cell responses are crucial for a protective anti-viral immunity [107]. Upon activation, CD4 T cells differentiate into TH1 cells that help activate cytotoxic CD8 T-cells and secrete IFN-γ [108]. On the other hand, TH2 response, characterized by a different cytokines signature (IL-4) causes immunopathology in many viral infections. Activated CD8 T cells release cytotoxic granules containing perforin, granzymes, and granulysin to induce the apoptosis in virally infected cells. In SARS-CoV-1, CD4 and CD8 T cell producing IFN-γ and TNF-α were found to be significantly increased in severe cases compared to mild–moderate cases [109].

Moreover, an increase in TH2 cytokine levels was noted in the sera of patients with a fatal infection. Chemokines (IL-8, MCP-1, IP-10), inflammatory cytokines, and TH1 cytokines (IFN-γ, IL-1, IL-6) were found to be elevated for 2 weeks after disease onset [110]. Zhou et al. recently showed that in COVID-19 patients, pathogenic TH1 cells secreting GM-CSF and CD14+CD16+ inflammatory monocytes secreting IL-6 are two cell populations that may infiltrate the lungs, causing severe pulmonary damage [111]. This suggests that GM-CSF or IL-6 antagonizing antibodies may be an effective treatment to reduce the inflammation and cytokine storm. Recovering SARS-CoV-2 patients develop N, RBD, and main protease specific T-cell response that appears to subside in almost all follow-up patients [105].

Interestingly, the levels of NAbs were significantly correlated to the numbers of N-specific T-cells, indicating that efficient humoral and cellular responses may be required for viral clearance [82]. S-specific CD4 T cells were found in 83% of SARS-CoV-2 and in 34% of seronegative healthy donors [112]. T-cells isolated from COVID-19 patients could target both the C and N-terminal of the S protein, In contrast, those isolated from healthy donors exclusively targeted the C-terminal (of higher homology to the common cold hCoVs, and not containing the RBD). Moreover, COVID-19 patient-isolated T-cells expressed higher CD38 and HLA-DR levels compared to healthy donors, indicating their recent activation status. Pathologic immune cell infiltration was identified in fatal cases of COVID-19. Image mass cytometry of post-mortem lung autopsy shows infiltration of CD4 T-cells, CD16 and CD107A NK cells, CD68 macrophages, CD11b and CD16 neutrophils [113]. Neutrophils involved in hyperinflammation and neutrophil extracellular traps (NETs) [114]. NETs have been previously described as important factors in tissue damage from inflammatory diseases and are used to ensnare invading pathogens. In COVID-19, the neutrophil-to-lymphocyte ratio has demonstrated enhanced inflammatory response in patients, contributing to ARDS [115]. The role of neutrophil-associated damage can be attributed to the high levels of NETs that were shown to be increased in tracheal aspirate and plasma from patients with severe COVID-19 and increased neutrophil-to-lymphocyte ratio [116]. Specifically, these patients produced more concentrations of NETs. Treatments targeting NET production from neutrophils could significantly reduce the collateral damage caused by neutrophil-driven hyperinflammation. This approach could decrease the severity of the disease, possibly forestalling the need for mechanical ventilation. Such drugs that target NET production include inhibitors of the precursor molecules needed for NET formation, such as gasdermin D [117] and PAD4 [118].

It has been recently proposed that SARS-CoV-2 may be able to infect TH cells by the interaction between S protein and CD4 [119]. SARS-CoV-2 infected TH cells secrete higher levels of IL-10 and lower proinflammatory cytokines e.g., IFN-γ and IL-17, which is more pronounced in severe cases than in moderate cases or in healthy individuals. The ex vivo stimulation of convalescent peripheral blood mononuclear cells (PBMCs) using SARS-CoV-2 peptides led to the expansion of T-cells, including two HLA-restricted CD8 T cell population targeting the spike protein (269–277) and Orf1ab3183–3191 [120]. Although all CD8 T cells expressed granzyme B or/and perforin, the A2/S269+CD8+ T cells lacked the expression of activation markers: HLA-DR, CD38, CD71, and PD-1. Furthermore, the frequencies of A2/S269+CD8+ T cells were lower than those specific to other infections (e.g., influenza) and were skewed toward a stem cell, naïve, and a central memory phenotype with a lack of effector memory T cells. This may explain why some patients may fail to elicit a protective immune response upon reinfection [121].

Furthermore, it has been recently observed that patients who were infected with SARS-CoV-1 in 2003 had memory T-cell responses to both SARS-CoV-1 and SARS-CoV-2 antigens 17 years post-infection. Individuals that possessed memory T cell responses against coronaviral nucleoprotein and NsPs 7 and 13 were shown to have less pathology to SARS-CoV-2 infection. Participants were screened, and it was revealed that some individuals that had no record of SARS-CoV-1 or SARS-CoV-2 infection possessed memory T cells that were capable of eliciting a protective immune response against SARS-CoV-2, raising the question of the exposure origin of this protective response [122]. Given that multiple coronavirus strains such as 229E, OC43, and HUK1 circulate globally as common cold viruses, prior exposure to these strains may result in protective T memory cell responses that could be effectively resist severe SARS-CoV-2 infection. This attribution can be made regarding the homology between these viruses at the conserved domains and catalytic regions of the nucleoprotein, NsP7, and 13, as robustly analyzed by Le Bert et al. [122]. These data are striking and may serve to help better clarify the discrepancies between the severely ill, with the mild and asymptomatic cases and could help inform future vaccine and therapeutic design against SARS-CoV-2.

## 4. Vaccine Development for SARS-CoV-2

Based on the severity of COVID-19, paired with the viral R_0_ of 2.2 (one infected individual infecting 2.2 others), and a mean incubation period of approximately one week, effective vaccines are of utmost need [123]. Vaccines are designed to train the immune system to recognize and attack the target virus as it encounters the host’s innate immune system. Upon producing a sufficient neutralizing antibody titer, an individual cannot be efficiently infected with the virus and the transmission cycle is broken, establishing the beginnings of herd immunity. The emergency use authorization by the FDA of the first two vaccines using mRNA encoded SARS-CoV-2 spike delivered via nanoparticle, followed by a third in the United Kingdom, using an adenoviral vector displaying SARS-CoV-2 spike. Many groups worldwide are still in the process of the vaccine development, which is usually under the strict scrutiny of phase I, II, and III clinical trials before reaching the clinic. Various research organizations and biotech/pharmaceutical corporations are collaborating with governments and global agencies such as NIH (National Institute of Health) and CEPI (Coalition for Epidemic Preparedness) to address this global pandemic in terms of vaccine research, development, and implementation.

### 4.1. Whole Virus Vaccine

The major attributes of the classical whole virus or live-attenuated virus vaccine is its elevated immunogenicity and long-lasting immune response apart from their safety issues, particularly in the immunocompromised. Although SARS-CoV-2 bears 89% similarity with the original SARS coronavirus, based on their severity of infection, critical safety concerns exist post whole/live attenuated virus immunization [124]. However, companies such as Johnson and Johnson (Janssen’s AdVac^®^ adenoviral vector & PER.C6 technology) and Codagenix in collaboration with the Serum Institute of India are developing these kinds of vaccines for SARS-CoV-2 [125]. AstraZeneca was recently granted emergency use authorization of its vaccine AZD 1222, which is an attenuated chimpanzee-based adenoviral vector expressing SARS-CoV-2 spike, in the United Kingdom. Clinical trial results demonstrated that the vaccine was well tolerated, with 70% efficacy after two doses were administered within a month [126]. In the United States, stage 3 clinical trials for this vaccine are in the recruitment stage, aiming for 40,000 participants [127].

### 4.2. Recombinant Protein Subunit Vaccines

SARS-CoV-2 Spike glycoprotein (crucial for viral entry into the host cell) encoding vaccines are being developed by many groups such as Clover Biopharmaceuticals, Novavax, and the University of Queensland by using S-trimer recombinant protein Trimer-Tag, recombinant nanoparticle, and molecular clamp technology, respectively. One of the most remarkable vaccine candidates in Phase I clinical trial is ChAdOx1, a nonreplicating chimpanzee adenovirus vector that is engineered to encode the spike protein of SARS-CoV-2. This vaccine results from the collaborative effort between the Oxford University Vaccine Group’s clinical teams and the University’s Jenner Institute including 510 volunteers aged 18–55 years. RBD vaccines formulated with alum have been developed by the University of Texas Medical Branch in collaboration with New York Blood Center as subunit protein vaccines. Vaxart, Inc., a clinical-stage biotechnology company, recently announced the development of oral recombinant vaccines based on its VAAST^TM^ platform in agreement with Emergent BioSolutions Inc. (“Emergent”) for development services in preparation for the cGMP production of the Vaxart vaccine. Their vaccine constructs consist of different coronavirus antigen combinations that are supposed to be initiating clinical trials soon.

### 4.3. Nucleic Acid Vaccines

DNA-based vaccine directly induces a plasmid for encoding the antigen of interest against which the immune response leads to the production of the target antigen in the patient’s cells. Entos Pharmaceuticals’ developed Fusogenix (a proprietary proteo-lipid vehicle (PLV), a nanomedicine-based platform for various SARS-CoV-2 antigen-encoding DNA vaccines for patient protection. Among the RNA vaccines, an initiative has been undertaken by the Imperial College London’s department of infectious disease to trigger muscle cells to produce the SARS-CoV-2 spike protein. This self-amplifying RNA (saRNA) vaccine will stimulate the immune system to generate neutralizing antibodies against the virus. Furthermore, the mRNA vaccine candidate BNT162b1, which encodes for the SARS-CoV-2 receptor–binding domain, and BNT162b2, which encodes for SARS-CoV-2 stabilized prefusion, full-length spike, were shown to be well-tolerated and safe in stage I clinical trials, producing a robust antibody response [128].

Other ongoing work under the global “Lightspeed” initiative for COVID-19 vaccine development involves multinational biopharma companies such as Pfizer and BioNTech. These companies have four vaccine candidates in lipid nanoparticle (LNP) formulation that are undergoing clinical evaluation in Germany. Two of the candidates are modified nucleoside mRNA (modRNA), one is uRNA (uridine-mRNA), and another is saRNA. These candidates utilize two encoded larger spike protein recombinants. The other two candidates consist of a smaller optimized RBD of the spike protein, which can inactivate SARS-CoV-2 by eliciting neutralizing antibody production [129,130]. Even more recently, Pfizer and BioNTech announced a 90% protective antibody response against SARS-CoV-2 in stage III clinical trials of their liposome nanoparticle mRNA vaccine. The phase III trial enrolled 43,538 participants, with 42% having diverse backgrounds [131]. Importantly, this vaccine has been granted FDA approval was the first vaccine authorized for use against SARS-CoV-2 in the United States. However, two doses are recommended within one month for the greatest efficacy to be achieved by the vaccine. Additionally, Moderna has received phase III clinical trial results, showing 94.5% efficacy in generating protective immunity by using mRNA as well, and also requires two doses of the vaccine. This formulation was also recently FDA approved for use in the United States. Furthermore, Inovio Pharmaceuticals is testing a MERS DNA vaccine on 40 volunteers, and the University of Queensland in Australia is investigating viral proteins derived from cell culture, which are undergoing preclinical testing. In the Netherlands and Australia, some clinical trials with TB vaccines are underway. Other pharmaceutical companies such as Johnson & Johnson, CureVac, and Sanofi are working on their own vaccine platforms. There are hundreds of vaccines that are under investigation; a select list of vaccines is presented in Table 1. While all of this vaccine research is encouraging, two vaccines, Pfizer-BioNTech and Moderna, have been authorized in many Western countries [132]. However, the fact that each of these vaccines is given in two doses presents a logistical problem, as mass vaccination is most effective by way of a single dose. Furthermore, the Pfizer vaccine requires −80 °C cold storage for the product’s stability, thus presenting a logistical hurdle, compared to Moderna’s more modest −20 °C for stability. In contrast to these RNA vaccines, the AstraZeneca vaccine is a single-dose adenoviral vaccine that uses standard cold-chain storage, and hence, it can be used more broadly worldwide [133]. Notably, more research is needed that is focused on increasing the immunogenicity of vaccines produced against SARS-CoV-2 and their associated emerging variant strains [133,134,135].

## 5. Immunotherapies for COVID-19

### 5.1. Cytokine Therapies

Interferons are a group of cytokines that upon binding to their cognate interferon receptors activate the JAK–STAT pathway and the transcription of ISG. As a result, an anti-viral response is produced by inducing viral RNA degradation, inhibition of viral transcription, vial protein synthesis, and induction of infected cell apoptosis [136]. The antagonism of IFN response leads to unchecked viral replication, an accumulation of PAMPs, and pathogenic inflammatory response [137]. The delayed IFN response in SARS-CoV-1 and MERS increases the recruitment of pathogenic inflammatory monocytes, cytokine/chemokine levels, and defective anti-viral T cell immunity in infected mice [138,139,140].

Consequently, the use of IFN for the treatment of hCoV infection was explored. In vivo administration of prophylactic pegylated IFN-α administration to SARS-CoV-1 infected macaques reduced viral replication, viral antigen expression, and lung damage [141]. JAK-1 phosphorylation, protein kinase C delta, and the MAP kinase p38 MAPK are essential for IFN-mediated protection against the SARS-related murine MHV-1 infection in vitro [142]. These studies suggest that IFN-I may be an effective treatment in the early stages of SARS-CoV-2, while in later stages, anti-IFN drugs could be useful to treat the lung immunopathology [143]. Conversely, investigations into Type 3-IFN, such as IFN-λ, have been demonstrated as having adverse effects upon the host by disrupting the lung epithelial barrier upon viral recognition of IFN-λ produced by dendritic cells in the lung in response to viral RNA inducing barrier damage and thus increasing the susceptibility of lethal bacterial infections and pneumonia [144]. This highlights a degree of the immunopathology caused by SARS-CoV-2 infection.

Another factor of interest in fighting the immunopathology caused by SARS-CoV-2 is IL-6. This specific interleukin has been used as a prognostic marker for a poor outcome as it relates to COVID-19 [145]. The upregulation of IL-6 during SARS-CoV-2 infection is attributed the viral NsPs, particularly NsP10 inhibition of NKRF, which regulates NF-kB suppression [145]. From this observation, IL-6 inhibition may be beneficial for patients with severe COVID-19. Tocilizumab, an anti-IL-6 receptor antibody, was investigated for its role in reducing COVID-19 IL-6-associated pathology [146,147]. Tocilizumab was found to be an effective treatment option for patients suffering from COVID-19, especially those who may be predisposed to having a CSS [147]. However, this result is still controversial, as one school of thought dismisses the notion of CSS because of the lack of a clear definition of this syndrome. Another school of thought is based on the observation that the median IL-6 levels are relatively low in patients with COVID-19, suggesting that the disease as a hypoinflammatory vasculopathy, rather than by hypercytokinemia. Thus, the potential of anti-IL-6 therapy as a therapeutic modality in treating severe COVID-19 requires further investigation.

### 5.2. Therapies for SARS-CoV-2/Coronavirus Infection

Currently, treatment options for coronavirus infection are limited and only marginally effective. Treatments being tested include nucleoside analogs (e.g., Remdesivir), anti-inflammatory drugs, RNA synthesis inhibitors, and inhibitors of viral fusion such as EK1 peptide [148,149]. Remdesivir received the FDA’s emergency use authorization; however, there are concerns about the drug’s efficacy after the acute period. Meta-analysis showed that 10 days of treatment increased the recovery rate by 50% on day 14 in severe COVID-19 patients (RR = 1.5, 95%CI = 1.33–1.7). Furthermore, on day 28, there was an additional increase of 14% in both moderate and severe COVID-19 patient recovery (RR = 1.14, 95%CI = 1.06–1.22). Importantly, treatment decreased mortality rate on day 14 by 36% in all patients (RR = 0.64, 95%CI = 0.45–0.92) but was not observed on day 28 (RR = 1.05, 95%CI = 0.56–1.97) [150]. Therefore, given the modest response rate to Remdesivir, other novel therapies with increased effectiveness in controlling SARS-CoV-2 pathogenicity are desperately needed. The development of peptide therapies to block viral entry into host cells has gained interest in recent years since Enfuvirtide, an inhibitor of HIV gp41, was approved for clinical use in 2004 [151]. In general, peptide drugs have high specificity and low toxicity, making them an attractive alternative to small molecule inhibitors [152]. Their disadvantages include low in vivo stability and bioavailability issues, which will need to be overcome before their use in the clinic. Methods to overcome these challenges include chemical modifications and nanoparticle formulations, but more work will be required in this area [153].

The first step to developing a peptide fusion inhibitor is determining the target. Peptide interactions with the spike protein, ACE2, or cellular proteases could alter the conformation of these proteins, inhibiting their activity and therefore prevent viral entry. Several approaches have been used to discover/design peptides that have the desired inhibitory interactions including high-throughput screening, structure-based design using sequence analysis and crystallography, prediction of hydrophobic interactions based on hydrophobicity score (Wimley and White interfacial hydrophobicity scale), and homology to hydrophobic viral protein domains [154,155,156].

Since the spike RBD has a high mutation rate, recent work has been focused on the more highly conserved HR1 and HR2 domains [149]. Several groups have designed peptides homologous to the spike protein, aiming to interrupt inter-domain interactions that occur during fusion [154,155,156]. This has led to the development of EK1, a peptide derived from the HR2 domain of hCoV-OC43, which forms a stable six-helix structure upon interacting with HR1, thus blocking its binding to viral HR2 and blocking viral fusion [149]. Furthermore, EK1 treatment has a protective effect in a mouse model of both hCoV and MERS-CoV, demonstrating its utility in vivo [157]. However, potency has historically been a challenge for peptide drugs, and subsequent studies suggest that EK1 may require modifications such as lipidation to increase potency, as it is likely to have limited endosomal uptake [149,157].

In addition to viral homology, anti-fusion peptides have also been designed using homology to host proteins. Specifically, a peptide with homology to host β-defensins (P9) has been shown to have a broad-spectrum antiviral activity against several strains of influenza and coronavirus [158]. β-defensins are proteins secreted by epithelial cells as a part of their innate immune response and bind to viral membranes or glycoproteins to reduce viral entry and replication. The P9 peptide, upon binding to viral glycoproteins, is taken up into endosomes, where it prevents the acidification necessary for the cleavage of fusion proteins by proteases. However, it is unclear whether P9 can inhibit SARS-CoV-2 fusion via the cell surface route mediated by host serine protease TMPRSS2.

## 6. Variants of SARS-CoV-2

The emergence of variants of SARS-CoV-2 has caused great international concern and alarm. One of the original mutations to gain recognition was the D614G mutation within the spike protein, which was shown to increase infectivity [159]. Another variant described by a mutation in its spike protein, L452R, was first detected in Denmark in March 2020 and was recently implicated in California’s significant outbreaks since November 2020 [160]. Three additional variants of great concern have been reported out of the United Kingdom, South Africa, and Brazil with higher than average mutations on the receptor-binding domains and demonstrated increases in infectivity and possible reinfection [161,162]. However, definitive increases in hospitalization rates and death associated with these variants are currently under investigation.

### 6.1. United Kingdom Variant B.1.1.7

The United Kingdom variant, known as 20I/501Y.V1 (synonyms: VOC 202012/01, or B.1.1.7), emerged around September 2020 with an atypically large number of mutations, particularly within RBD including N501Y, P681H proximal to the furin cleavage site S1/S2; position 69/70 deletion resulting in a conformation change in the spike, and ORF8 stop codon insertion, Q27 with an unknown function [163]. Similar to what has been previously seen in SARS-CoV-1, a deleterious 29 nucleotide deletion was found in ORF8, which plays a role in immune evasion and interferon suppression of SARS-CoV-2 and is considered one of the least conserved proteins among coronaviruses [164,165]. Recently, B.1.1.7 has been detected in several other countries including the United States and Canada. Alarmingly, this variant is shown to have increased transmissibility associated with a higher affinity to ACE2. Given the widespread dissemination of this variant, robust research efforts to investigate any heightened lethality associated with this particular variant. However, this measure is challenging to assess, given the heightened increase in infectivity, which correlates to a higher total death count, but not necessarily a higher rate. The New and Emerging Respiratory Virus Threats Advisory Group of the UK recently published a document discussing B.1.1.7 increased disease severity [166]. The advisory group agrees that B.1.1.7 demonstrates increased transmissibility compared to other SARS-CoV-2 and has rapidly become the dominant variant in much of the United Kingdom. The initial assessment of disease severity via a matched case-controlled study revealed no significant risk of increased hospitalization or death of people infected with B.1.1.7 variant compared with other variants [167]. However, there have been several new analyses that have reported increased disease severity in individuals infected with B.1.1.7 compared with those infected with non-variants of concern. Public Health England has recently updated a matched cohort analysis which reported a death risk ratio for B.1.1.7 infected people compared to non-Variant of Concern of 1.65 (95%CI 1.21–2.25) [166]. Admittedly, there are limitations to these datasets based on transmission setting, potential bias in case assessment, representativeness of death data, and power. However, these analyses suggest that infection with B.1.1.7 could be associated with an increased risk of hospitalization and death compared to infection with non-variant viruses. This conflicting and preliminary information underscores the dire need for greater epidemiological, virological, and pathological characterization of this variant in a robust scientific setting.

### 6.2. South Africa Variant B.1.351

The South African variant known as 20H/501Y.V2 or simply B.1.351 shares some mutations with B.1.1.7; however, it emerged independently. B.1.351 was first discovered in Nelson Mandela Bay, South Africa in October 2020, and cases have now been seen outside of the country [168]. This variant appeared to be the dominant variety in Zambia as of December 2020. Mutations in the spike protein include K417T, E484K, and N501Y, but it does not contain the deletion at 69/70 that is present in the UK variant. E484K, present in the RBD of Spike, has been shown to confer resistance to some polyclonal and monoclonal antibodies [168]. A recent study demonstrated that convalescent plasma was incapable of neutralizing this SARS-CoV-2 variant, raising further concern for reinfection [169]. It has not yet been fully explored whether or not this emerging strain shows heightened disease.

### 6.3. Brazil Variant P.1

The Brazilian variant, P.1, was first detected in Japan from four travelers from Brazil that were sampled during an arrival screening in Tokyo. This particular variant has three deletions and 17 unique mutations, three of which are present in the RBD of spike: K417T, E484K, and N501Y [170]. Recent evidence suggests that these mutations may impact the transmissibility and antigenic profile. These changes could affect antibodies produced from a resolved infection or through vaccination that would bind and neutralize the spike protein. A recent study in Manaus, Brazil, identified that the P.1 variant was in 42% of the sequenced specimens in late December 2020 [170]. It had been previously estimated that 75% of the region’s inhabitants had been infected with SARS-CoV-2 by October 2020. Alarmingly, the area has experienced a surge in cases since mid-December 2020, which raised a grave concern for increased transmissibility and or re-infection of individuals by this unique variant. Researchers are diligently working to discover more about these SARS-CoV-2 variants to understand better how these mutations affect transmission and analyze the effectiveness of currently authorized vaccines, which may already require an update. Currently, there is very little evidence investigating if these variants cause increased illness or risk of death. However, new information about the characteristics of these variants is quickly emerging. Identifying these variants indeed underscores the need for increased sequencing and surveillance of emerging SARS-CoV-2 variants, so preparations can be made to alter approaches that may not cover the newly emerging variants of this virus.

## 7. Conclusions and Future Perspectives

Emerging zoonotic coronaviruses are a significant issue of international public health concern. With the emergence of the novel coronavirus, SARS-CoV-2, global public health structures were rapidly overwhelmed with COVID-19 cases, in part due to a lack of effective therapies against the virus and a lack of understanding of viral pathology. The current emergence of variant strains with increased transmissibility associated with higher affinity for ACE2 puts a further strain on healthcare resources to this day. In this review, all three concerns were sought to be addressed, emphasizing immunopathology caused by SARS-CoV-2 and other Coronaviridae members. Coronavirus strains with regions of homology to SARS-CoV-2 could elucidate potentially conserved mechanisms of action, as well as how this information contributes to the understanding of the emerging variants of SARS-CoV-2. Roles of the non-structural proteins were explored for their ability to promote immune dysregulation that could be responsible, at least in part, for the severe pathology caused by this virus. Alternate cellular binding domains, CD147, NRP1, and myeloid CLRs were also described as potential alternatives to ACE2 to explain the lack of tissue tropism seen with SARS-CoV-2.

The global effort to apply existing treatments, develop new ones, and create vaccines against SARS-CoV-2 is a major international priority that has shown some success with the authorization of several vaccines for use. This review covers therapeutics under active investigation and the list of SARS-CoV-2 vaccines in preclinical and clinical development and their intended applications. However, given the high mutation rate of this virus and SARS-CoV-2 global genome tracking from GISAID (https://www.gisaid.org/ (accessed on 22 January 2021)), it is worth noting that vaccines and therapeutics may have a short period of efficacy, as is being demonstrated with antibody resistance exhibited by some of the variant strains. Hence, host factors that may be therapeutically targeted must also be considered to prevent the severe pathologies caused by the viral proteins-induced immune dysregulation. It was previously reported that NF-kB inhibition increased the lifespan of mice infected with SARS-CoV-1, implicating the virus in aberrant inflammation associated with improper signaling [72]. Other targets, such as NETs, could limit collateral damage from host hyper inflammation in response to SARS-CoV-2, variants, and otherwise. Other advantages of targeting host factors are that they are far more constant than viral factors, thus promoting the possibility of long-term immunity through greater immune regulation. For example, the aerosolization of corticosteroids may help reduce the aberrant inflammation caused by the virus, preserving the lung architecture from further damage, but this also requires further study [171,172,173]. In addition, a randomized clinical trial in the United Kingdom showed a positive effect for the steroid dexamethasone for treating COVID-19 [174]. This is significant, as it is the first major study that corroborates anecdotal evidence suggesting that steroids may ablate the cytokine pathologies observed in COVID-19. Other considerations include combination therapies, targeting both the virus and the host in extreme disease cases; however, greater characterization of SARS-CoV-2 is needed, particularly in identifying highly conserved domains whose inhibition might yield a potent antiviral effect.

Based on the successes and failures of other anti-viral vaccines, it is difficult to predict whether and to what extent the COVID-19 vaccines will provide protection to and reduce mortality of the frail elderly with immunosenescent immune systems. Clinical trial data on the mRNA vaccines have shown robust antibody responses in this group. Still, the duration of immunity is unclear, and booster vaccinations might likely be needed annually. Even in healthy individuals, SARS-CoV-2 antibodies appear to have a short duration, with titers declining at an average of about four-fold at one to four months after the onset of symptoms [175]. However, the capacity for infected humans to generate effective neutralizing antibodies against the RBD has been observed, but it is inconsistent from patient to patient [176,177]. An additional challenge that is currently under intense investigation is the impact the variant strains would have on the effectiveness of vaccines and therapeutics, as convalescent sera from the South African variant was shown to be incapable of neutralizing the virus [169].

The emergence of effective vaccines is promising, but the SARS-CoV-2 is still rapidly mutating, and there is mounting evidence supporting the possibility of immune escape from a novel mutation. Meanwhile, the treatment approaches that include anti-viral and anti-host-based mechanisms may lead to better pre-and post-exposure prophylaxis and effective treatments for SARS-CoV-2.

## Figures and Tables

**Table 1:** Selection of SARS-CoV-2 vaccines authorized for use, as well as others in preclinical and clinical development, their intended applications and technologies.

Name of Vaccine	Organization
**Nucleic Acid Vaccines**	
Fusogenix	Entos Pharmaceuticals
self-amplifying RNA (saRNA	Imperial College London
modified mRNA (modRNA): uRNA (uridine-mRNA) Spike, RBD	Pfizer
modified mRNA (modRNA): saRNA: spike, RBD	BioNTech
mRNA	Moderna
DNA vaccine	Inovio Pharm.
viral proteins in cell culture	Queensland in Australia
TB vaccines	Netherlands and Australia
**Live-Attenuated/Whole Virus Vaccines**	
Codegenix & dia	Serum Institute of In
AdVac adenoviral vector	Janssen
PER.C6 technology	Johnson & Johnson
AZD 1222 adenoviral vector	AstraZeneca
**Recombinant Protein Subunit Vaccines**	
S-trimer recombinant protein Trimer-Tag	Clover Biopharmaceuticals
Recombinant nanoparticle	Novavax
Molecular clamp technology	University of Queensland
Phase I clinical trial of ChAdOx1, a nonreplicating chimpanzee adenovirus vector, engineered to encode the spike protein	Oxford University and Jenner Institute
RBD formulated with alum	University of Texas Medical Branch and New York Blood Center
Oral recombinant vaccine using VAAST^TM^ platform	Vaxart, Inc.

## Data Availability

No new data were created or analyzed in this study. Data sharing is not applicable to this article. All data discussed in this review is cited and can be found in the references section of this manuscript.
